# Experimenting with a bundled payment system for diabetes care in the Netherlands: The first tangible effects

**Published:** 2011-11-18

**Authors:** Thomas Czypionka

**Affiliations:** Institute for Advanced Studies Vienna, Stumpergasse 56, 1060 Vienna, Austria E-mail: thomas.czypionka@ihs.ac.at

Payment systems exert considerable influence on how healthcare is delivered to patients, and the question whether integration of care can be furthered by such a scheme is topical. The US have only recently promoted the idea of integration by introducing the idea of accountable care organisations, while the UK is moving toward ‘clinical commissioning’, the idea of fundholding by GPs reborn. The Dutch answer to the challenge of treating chronic diseases and how to ensure its efficient delivery is the idea of a bundled payment system, the ‘keten-DBC’ in Dutch, also known as chain-DTC in English. The first evaluation results for the chain-DTC system for diabetes is the topic of this book.

Chapter 1 gives a short and well-structured introduction to the matter. After laying out the basic facts about the Netherlands with respect to diabetes, the motivation for politicians and the political development that led to the introduction of the bundles payment system is given, alongside the setup for the evaluation presented later in the book. A shortcoming of this chapter, though, is the description of the bundled payment system itself. The authors should probably have given more detail about it at this point because as is, the reader has to wait until Chapter 3 to grasp what the bundled payment system encompasses.

Chapter 2 provides an introduction to the Dutch healthcare system on seven pages. While this is expendable for most readers, it might come in handy for those that are not so familiar with it. The rising interest in bundled payment systems throughout the world, being a reason why the report has been published in English in the first place, justifies the inclusion of the chapter.

Chapter 3 goes into the details of the evaluation results on forty pages. Seven key questions stood at the beginning of the evaluation, and are answered throughout this chapter, thereby providing a useful structure for the reader to easily understand the results. The seven questions, and thus also the headlines of the seven sub-chapters, are:

1. What are the basic premises of the bundled care model?
2. How are the diabetes care groups organised in practice?
3. What are the principal features of bundled payment schemes for diabetes?
4. How does the health care purchasing process work?
5. How is the work carried out?
6. What quality of care is provided by diabetes care groups at the end of the 12-month period?
7. How satisfied are the various stakeholders?

Patient records, patient questionnaires and semi-structured interviews with officials from care-groups, providers and insurance companies formed the basis of the evaluation, which monitored nine care groups for the duration of twelve months. From the initial ten groups one dropped out prematurely.

Although the reader expects the results of the evaluation of the bundled care system, the first research question is actually an explanation of the idea itself: A basic health care standard is defined by the Dutch Diabetes foundation in terms of activities necessary in the care of diabetes (but not type of provider). It provides the template of care that so - called care-groups will have to deliver. Insurance companies and care-groups negotiate a price per patient for the diabetes care included in this predefined bundle, and the care group, usually led by one or more GPs, is then responsible for sub-contracting all other necessary providers.

The next part of the chapter provides us with the answers to the second question, a first empirical glance on what organisational structures the care groups came up with. As the whole idea is based on a lot of entrepreneurial freedom, this indeed is an interesting insight. The authors give us a well-considered report, also addressing problems, like the probable irreconcilability in terms of accountablity when a subcontracted provider is also co-owner of the care group, thus making a contract with itself.

The third sub-chapter takes a closer look at the content of the package actually contracted with the ten care-groups and the fees charged. A brief but concise analysis compares the healthcare standard to the services provided by the examined care-groups, also raising the issue whether the healthcare standard defined is precise enough. Concerning the fees, the authors detect considerable variations and encounter the problem that, due to trade secrecy, actual costs were not provided by the care-groups to them, so no relation could be established between fees and actual costs. Subcontracts could only be evaluated in some cases, showing considerable variation.

In the fourth sub-chapter, some findings on the actual purchasing process are discussed. Two of the main issues raised are the lack of experience in the first year on all sides and the considerable negotiating power of the care-groups.

The fifth sub-chapter examines, mostly based on interviews, how care delivery itself was organised. The focus lies on the management of the care-group as well as the coordination of services. The problem of double payments (for the same service within the bundle and outside of it) is also discussed, as it is of concern to the insurance companies and requires more attention.

The sixth sub-chapter provides results concerning the quality of the care delivered. Process indicators like the share of patients receiving the necessary check-ups and (surrogate) outcome indicators like LDL-levels were used to assess the performance of the care-groups under the bundled payment scheme.

The final chapter collects experiences and opinions from care-groups, their patients, insurance companies and providers.

The fourth chapter provides the reader with a discussion of the research itself and its results as well as policy implications. Among other things, the short period of evaluation and the lack of information in some areas like cost information are seen as drawbacks, but a follow-up is already on the way. The discussion of the results themselves points out several issues that support the model, but also some elements that should be of concern to policy-makers. Points like these include the induction of demand by including mild cases to reap the full bundled payment for more people, the considerable regional market power of a care-group or the lack of quality indicators that reflect the integrated nature of the system. The care standards were not always unequivocal. In a third section, policy implications are addressed, and the authors criticize the speed of introduction of bundled payments for more chronic diseases as of January 2010, without the evidence created by the present report. Concern is raised by the authors that the implementation of bundled payments with healthcare standards as a basis will create considerable pressure from providers on the development of these guidelines, as through the bundling, they essentially become compulsory, and more care than needed might be included in the first place. Based on these findings, recommendations are made on improvements and areas for further research.

The book concludes with an extensive appendix, providing the reader with more detailed findings concerning the structure of care groups, the quality indicators used, the methodology and further details of the patient survey and two small chapters on issues of liability within the bundled payment system and a further analysis of the implications for market regulation. A reader from health-economics or health services research is recommended not to skip this chapter, as it contains an interesting and very relevant discussion of the problems raised by the purchasing power of the care-groups. It should probably have been included into the main text.

All in all, this book can be recommended to everyone who works in the field of integrated care, as it provides its readers with valuable input and evidence. Its chapters are in themselves well-structured, although some decisions on the structure of the overall text can be questioned, e.g. hiding a very relevant chapter in the appendix. Notwithstanding, the authors did a good job evaluating and summarising this new development, with an easy to read first part and an abundance of information to look up given in the appendices.

